# Extremely elevated alpha-fetoprotein due to acute exacerbation of chronic hepatitis B without malignancy: a case report

**DOI:** 10.1186/s13256-016-0915-6

**Published:** 2016-06-01

**Authors:** Young-min Yoon, Da-yeong Kang, Da-young Kim, Jun-won Seo, Hyun-Jong Lim, Hee-Jeong Lee, Sang-Gon Park

**Affiliations:** 1 Department of Internal Medicine, Chosun University Hospital, 365 Pilmun-daero, Dong-gu, Gwangju, 501-717 Republic of Korea; 2 Department of Medicine, Graduate School of Chosun University, 309 Pilmun-daero, Dong-gu, Gwangju, 501-717 Republic of Korea; 3 Department of Internal Medicine, Hemato-oncology, Chosun University Hospital, 365 Pilmun-daero, Dong-gu, Gwangju, 501-717 Republic of Korea

**Keywords:** α-Fetoprotein, Tumor markers, Hepatitis B, Hepatocellular carcinoma, Testicular cancer

## Abstract

**Background:**

Alpha-fetoprotein is produced by a variety of tumors such as hepatocellular carcinoma, hepatoblastoma, and germ cell tumors of the ovary and testes. However, we present a case of significantly elevated serum alpha-fetoprotein without evidence of malignant disease in a patient who is a carrier of chronic hepatitis B.

**Case presentation:**

A 60-year-old Korean man presented with markedly increased alpha-fetoprotein (2350 ng/mL; normal <5 ng/mL). Various diagnostic evaluations, including computed tomography of the abdomen and thorax and ultrasonography of the abdomen and testes, showed liver cirrhosis and mild splenomegaly; however, no mass was detected in the liver, testes, or other organs scanned. The laboratory findings showed elevated liver function, positivity for hepatitis B e antigen, and a marked increase in hepatitis B virus deoxyribonucleic acid copy number (>7 × 105 IU/mL). Our patient was diagnosed with acute exacerbation of chronic hepatitis B, and we presumed that this condition might be related to extremely elevated alpha-fetoprotein. When our patient was treated with entecavir, the serum alpha-fetoprotein level immediately decreased, in parallel with the hepatitis B virus deoxyribonucleic acid copy number.

**Conclusions:**

We report a rare case of extremely elevated alpha-fetoprotein due to acute exacerbation of chronic hepatitis B without any malignancy, and a decrease in this tumor marker simultaneous with a decrease in hepatitis B virus deoxyribonucleic acid copy number on entecavir treatment. This case report is important due to the rarity of the case; furthermore, it provides details of a diagnostic process for a variety of benign diseases and malignant tumors that should be considered in patients with elevated alpha-fetoprotein. Thus, we present a case report, along with a review, that will be helpful for diagnosis and treatment of patients with elevated alpha-fetoprotein.

## Background

Serum tumor markers (STMs) are widely used in clinical settings as effective indicators for screening of malignant tumors, diagnosis of tumor type, establishing a prognosis, analyzing treatment effects, and early detection of relapse. Although the roles of STMs are limited in many cases, owing to their varying sensitivity and specificity, when STM values are markedly elevated, the corresponding cancer is strongly suspected [1]. In particular, alpha-fetoprotein (α-FP) is a key marker in screening for liver cancer in high-risk patient groups [2, 3].

## Case presentation

A 60-year-old Korean man was admitted to our institution due to elevated α-FP discovered during a health screening examination. He had been diagnosed with chronic hepatitis B 6 years prior and received regular follow-up observations; however, he had not been prescribed any medication, as no abnormal findings associated with indicators of active hepatitis, cirrhosis, or liver cancer were discovered. In addition, our patient had not received an abdominal ultrasound examination or a blood test in the past 2 years.

Our patient showed a highly elevated α-FP level of 2350 ng/mL (normal; <5 ng/mL), along with elevated aspartate aminotransferase (AST), 201 U/L (5–40 U/L), alanine aminotransferase (ALT), 209 U/L (5–40 U/L), total bilirubin, 1.91 mg/dL (0.2–1.2 mg/dL), direct bilirubin, 1.3 mg/dL, alkaline phosphatase, 100 U/L (35–123 U/L), and gamma-glutamyl transpeptidase, (r-GTP) 256 U/L (15–73 U/L). Our patient had no history of taking herbal or traditional Korean medicines or western medicines, and no history of infection or travel.

Although our patient was considered to be at high risk for liver cancer because he was a carrier of chronic hepatitis B, no liver tumors were observed on the abdominal ultrasound image; however, splenomegaly accompanied by moderate cirrhosis was found. Interventional dynamic computed tomography (CT) was performed on his chest, abdomen, and pelvis for precision testing of the liver parenchyma and assessment of cancers that typically show elevated α-FP, such as testicular cancer and germ cell cancer, along with paraneoplastic syndrome, which also results in secretion of α-FP. However, the findings again indicated only cirrhosis, as no tumor or lymphadenopathy was observed in the liver parenchyma, chest, abdomen, or pelvis. Moreover, there were no specific findings from a precision urological physical examination and a testicular ultrasound scan. In addition, gastric endoscopy was performed to test for α-FP-secreting hepatoid gastric adenocarcinoma, a common gastric cancer in Korea that is known to have a poor prognosis; however, no malignancy was detected. Other tumor markers, such as beta-human chorionic gonadotropin (β-HCG) <1.20 mIU/mL (0–1.25 mIU/mL), protein induced by vitamin K absence or antagonist-II (PIVKA-II) 11 mAU (0–39 mAU), and carcinoembryonic antigen (CEA) 2.8 ng/mL (0–5 ng/mL), were all within the normal range.

Precision testing for hepatitis was performed because our patient had a history of chronic hepatitis B and showed elevated AST and ALT. The test results were hepatitis B surface antigen positive (HBsAg+), hepatitis B e antibody negative (HBsAb−), and hepatitis C virus antibody positive (HCV Ab−), and additional tests showed hepatitis B e antigen positive (HBeAg+) and hepatitis B e antibody negative (HBeAb−). Moreover, since our patient also showed a marked elevation in hepatitis B virus deoxyribonucleic acid (HBV DNA) copy number (>7 × 10^5^ IU/mL), he was diagnosed with acute exacerbation of chronic hepatitis B. Although it is extremely rare for α-FP to be elevated above 2000 ng/mL (normal; <5 ng/mL) due to acute hepatitis B, there was a case study reporting decreased α-FP following treatment for acute hepatitis B. Treatment for hepatitis B was administered first and a follow-up imaging test was scheduled for 2 months later.

**Fig. 1:**
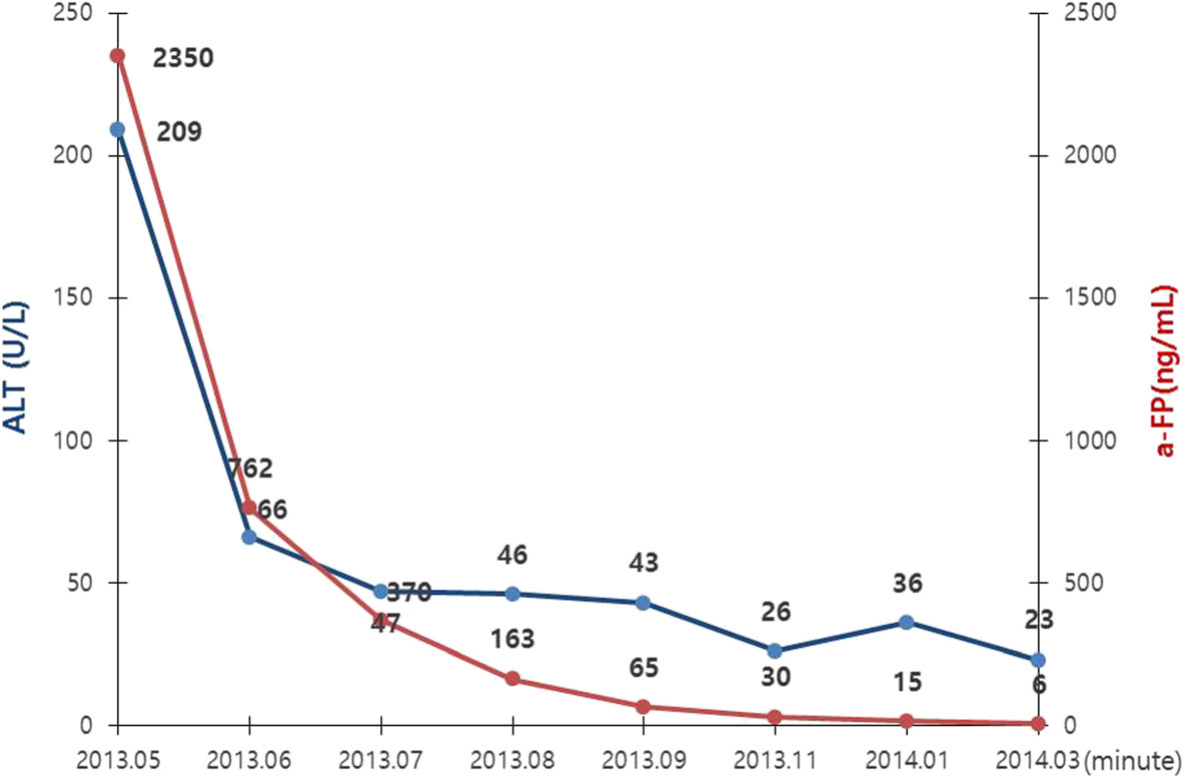
After taking the entecavir, α-fetoprotein and aspartate aminotransferase were gradually decreased

After 1 month of entecavir treatment in our patient, his AST/ALT and α-FP values (60/66 U/L and 762 ng/mL, respectively) significantly decreased. For AST/ALT and α-FP, these values further decreased to 53/47 U/L and 370 ng/mL, respectively, after 2 months, and to 39/36 U/L and 163 ng/mL, respectively, after 3 months. After treatment with entecavir, the HBV DNA copy number decreased to an immeasurable level after 3 months and α-FP was normalized after 11 months (Fig. 1). CT scans taken at 2 and 6 months of his chest, abdomen, and pelvis, revealed no tumor mass or lymphadenopathy in any solid organ, including his liver and testes. Currently, our patient is in the second year of follow-up examination, and normal levels of α-FP and HBV DNA copy number have been maintained, and no evidence of a malignant tumor has been found in any of the imaging tests (Fig. 1).

## Discussion

Tumor markers include various hormones, enzymes, and antigens secreted into the bloodstream by malignant tumors. Because these tumor markers can be used in simple blood tests to screen for malignant tumors, diagnose cancer type, provide a prognosis, analyze treatment effects, and provide early detection of relapse, they are in wide clinical use, despite their many limitations such as variations in sensitivity and specificity. Especially in people without signs or symptoms of tumors, assessing the levels of prostate-specific antigen (PSA) for prostate cancer, cancer antigen 125 (CA125) for ovarian cancer, and alpha-fetoprotein (α-FP) for hepatocellular carcinoma (HCC) is used as a minimally invasive screening test using blood samples [1].

Among these, α-FP is a glycoprotein secreted by fetal cells during the embryonic stage and in some malignant diseases. In 1944, Pederson first discovered a fetus-specific serum, and in 1964, Tatarino reported α-FP in the serum of a patient with hepatocytic cancer. Ruoslahti *et al*. purified human α-FP in 1971 and developed a radioimmunoassay technique to quantify it, which has since been used in clinical settings. Currently, α-FP is traditionally and widely used as a tumor marker for HCC. However, the sensitivity and specificity for using α-FP to detect HCC is only in the range of 41–65 % and 80–90 %, respectively, as serum α-FP is normal in up to 35 % of small hepatocellular carcinoma cases [2–4].

Several tools used alone or in combination with α-FP have been evaluated, as there are many different views regarding the use of α-FP as a biomarker. For example, a recent Japanese guideline indicates the combined use of tumor markers (α-FP >200 ng/mL, α-FP -L3 >15 %, or PIVKA-II >40 mAU/mL) for the diagnosis of HCC [5]. On the contrary, α-FP is considered to be a feasible tool for screening and early diagnosis in China as more than 60 % of HCC patients have an α-FP level of >400 ng/ml [6].

Although α-FP is widely known as a major marker for primary liver cancer, it is also known to increase with a variety of other diseases such as various malignant tumors, hepatic necrosis, acute hepatitis, colitis, and gynecological diseases [1]. Generally, when α-FP is elevated but no tumor can be detected through imaging, a regular follow-up observation is conducted with regular monitoring of progress. However, when the α-FP value is markedly elevated, as seen in the current case, additional imaging tests are performed on the liver because the possibility of malignant tumor cannot be excluded. Other cancers must be also considered; however, no report has been published outlining which cancers should be suspected or the extent to which an examination should be conducted [2].

As the name fetoprotein indicates, levels of this protein are elevated during pregnancy, but can also be elevated, along with ß-human chorionic gonadotropin (ß-HCG), in ovarian, endometrial, testicular, and extragonadal germ cell cancers, depending on gender. Therefore, in addition to testing for serum ß-HCG and imaging the uro-reproductive system, a close examination by a urologist or gynecologist is necessary. In particular, extragonadal germ cell tumors often develop in the retroperitoneum and mediastinum, whereas teratomas are observed in a variety of areas, and have been reported to become more frequent following lung cancer; hence, chest imaging is also required [1, 7]. In Korea, which has an especially high prevalence of gastric cancer, the prevalence of hepatoid adenocarcinoma of the stomach, which produces high levels of α-FP and looks similar to primary liver cancer, is known to be low (0.3–1 %). However, since its prognosis is known to be extremely poor, because of its high rate of metastasis to the liver and other organs before diagnosis, a gastroscopy may be necessary [8].

Few cases have been reported in which α-FP was elevated without any evidence of a malignant tumor; however, studies have reported values elevated to 2002 ng/mL in young patients with autoimmune hepatitis, and up to 371.51 ng/mL in adults with cavernous hemangioma [9, 10].

A case similar to the current case, in which α-FP was elevated to >2000 ng/mL due to acute exacerbation of hepatitis, but normalized following anti-viral therapy, as well as a few other cases have been reported internationally. However, cases like these are extremely rare, and even in the above-mentioned cases, the authors only discussed proof for the absence of liver cancer and the treatment process for acute hepatitis; they did not discuss ruling out other diseases that could have potentially increased α-FP [11–13].

## Conclusions

Here, we present a case of α-FP elevation due to acute exacerbation of chronic hepatitis without any evidence of a malignant tumor, along with a literature review.

This case report is important because of the rarity of the case, and provides details of a diagnostic process for benign diseases (acute hepatitis and hemangioma) and malignant tumors (testicular cancer, germ cell tumors, hepatocellular carcinoma, and gastric adenocarcinoma) that should be considered in patients with elevated α-FP. We believe that the findings presented in this report will be helpful for the diagnosis and treatment of patients with elevated α-FP.

## Abbreviations

α-FP: alpha-fetoprotein
ALT: alanine aminotransferase
AST: aspartate aminotransferase
ß-HCG: beta-human chorionic gonadotropin
CA125: cancer antigen125
CEA: carcinoembryonic antigen
CT: computed tomography
HBeAb: hepatitis B e antibody
HBeAg: hepatitis B e antigen
HBsAb: hepatitis B surface antibody
HBsAg: hepatitis B surface antigen
HBV DNA: hepatitis B virus deoxyribonucleic acid
HCC: hepatocellular carcinoma
HCV Ab: hepatitis C virus antibody
PIVKA-II: protein induced by vitamin K absence or antagonist-II
PSA: prostate-specific antigen
r-GTP: gamma-glutamyl transpeptidase
STMs: serum tumor markers
